# Comprehensive annotation and evolutionary insights into the canine (*Canis lupus familiaris*) antigen receptor loci

**DOI:** 10.1007/s00251-017-1028-0

**Published:** 2017-09-19

**Authors:** Jolyon Martin, Hannes Ponstingl, Marie-Paule Lefranc, Joy Archer, David Sargan, Allan Bradley

**Affiliations:** 1 Wellcome Trust Sanger Institute, Hinxton, UK; 2 University of Cambridge, Cambridge, UK; 3 University of Montpellier, CNRS, IGH UMR9002, Montpellier, France

**Keywords:** Canine, Antigen receptor, Immunoglobulin, T cell receptor

## Abstract

Dogs are an excellent model for human disease. For example, the treatment of canine lymphoma has been predictive of the human response to that treatment. However, an incomplete picture of canine (*Canis lupus familiaris*) immunoglobulin (IG) and T cell receptor (TR)—or antigen receptor (AR)—gene loci has restricted their utility. This work advances the annotation of the canine AR loci and looks into breed-specific features of the loci. Bioinformatic analysis of unbiased RNA sequence data was used to complete the annotation of the canine AR genes. This annotation was used to query 107 whole genome sequences from 19 breeds and identified over 5500 alleles across the 550 genes of the seven AR loci: the IG heavy, kappa, and lambda loci; and the TR alpha, beta, gamma, and delta loci. Of note was the discovery that half of the IGK variable (V) genes were located downstream of, and inverted with respect to, the rest of the locus. Analysis of the germline sequences of all the AR V genes identified greater conservation between dog and human than mouse with either. This work brings our understanding of the genetic diversity and expression of AR in dogs to the same completeness as that of mice and men, making it the third species to have all AR loci comprehensively and accurately annotated. The large number of germline sequences serves as a reference for future studies, and has allowed statistically powerful conclusions to be drawn on the pressures that have shaped these loci.

## Introduction

It is imperative for the body to be able to detect, recognise, and respond to pathogens. Given the variety and changeable nature of pathogens, it is neither practical nor possible to pre-encode the means to recognise every potential pathogenic agent. The immunoglobulin superfamily-based adaptive immune system (AIS) emerged as a solution to this problem 500 million years ago in jawed fish (Flajnik and Kasahara 2010). The AIS comprises of three families of lymphocyte cell surface receptors, those of the major histocompatibility (MH) proteins, the T cell receptor (TR), and the immunoglobulin (IG).

The MH diverged from the IG and TR further back in evolutionary time, and the split predates the co-opting of the recombination-activating gene (RAG) transposon that is at the heart of the recombinatorial diversity of the IG and TR, collectively known as antigen receptor (AR) genes (Nei 2005; Flajnik and Kasahara 2010). Recombination signal (RS) sequences flank the variable (V), diversity (D), and joining (J) genes of these loci and allow these genes to be recombined within B or T cells in order to generate the diversity and pattern recognition ability of their surface receptors. The study of the AR loci has not only furthered human medicine, but as more species are studied it has broadened into research in comparative genomics and evolution, as well as potential applications in veterinary medicine. Within the dog, the T cell receptor beta (TRB) and gamma (TRG) and the immunoglobulin heavy (IGH) loci have previously been annotated (Lefranc et al. 2009; Massari et al. 2009; Matiasovic et al. 2009; Bao et al. 2010; Mineccia et al. 2012). However, to date no annotation has been publically available for either immunoglobulin light locus, nor for the T cell receptor alpha and delta (TRA/TRD) locus.

In this study, the canine (*Canis lupus familiaris*) immunoglobulin kappa and lambda (IGK, IGL) as well as the TRA/TRD loci were annotated, and the annotation of the IGH locus was updated. Whole genome sequence data from over 100 dogs was used to identify 5000 non-reference alleles, and these shed light on the evolutionary pressures that have shaped these loci. Cross-species comparisons have lent further insight and confirmed the dog as a more faithful immune model of humans.

## Materials and methods

### Bioinformatic annotation

The loci were initially annotated following a method similar to Das and colleagues and using equivalent principles to the algorithm of Olivieri and colleagues (Das et al. 2008; Olivieri et al. 2013). Broad chromosomal regions were identified by interrogating the canine reference genome (CanFam3.1—GenBank Assembly ID: GCA_000002285.2) with genes from a given mouse or human locus. Once a likely region, as determined by a clustering of hits from both V and C genes, was identified, it was locally searched for canine genes. Consensuses from conserved human and mouse sequences such as the RS, the leader exon, and parts of the framework regions were used to search for canine AR genes. As more canine genes were annotated, their consensuses in turn were used to search for further genes. This bioinformatic annotation was then compared to the regions to which the RNA-seq reads aligned in order to refine it further.

### Dogs

Peripheral blood samples were secured from 26 dogs (Table 1). The samples were unused clinical excess of veterinarian-mandated blood draws from patients seen at the veterinary hospital of the University of Cambridge. This study received prior approval from the ethics committee of the Veterinary School of the University of Cambridge.

**Table 1:** Breed, sex, and age of the dogs used for annotation. M = male, MN = male neutered, F = female, FN = female neutered

Breed	Sex	Age
American bulldog	FN	4 years 2 months
Beagle	F	4 years
Border Collie	FN	7 years 3 months
Border Collie	MN	6 years 11 months
Boxer	MN	5 years 3 months
Bull Mastiff	FN	8 years 9 months
Bernese Mountain Dog	MN	7 years 3 months
Cavalier King Charles Spaniel	FN	10 years 1 months
Cocker Spaniel	FN	12 years 5 months
Cocker Spaniel	M	4 years 9 months
Cocker Spaniel	MN	3 years 11 months
Cross Breed	MN	7 years 4 months
Flat Coat Retriever	MN	6 years 7 months
Flat Coat Retriever	FN	10 years
Jack Russel Terrier	FN	6 years 7 months
Jack Russel Terrier	MN	13 years 3 months
Jack Russel Terrier	MN	4 years 4 months
Labrador	FN	8 years
Labrador	FN	6 years 9 months
Labrador	MN	9 years 2 months
Labrador	MN	6 years 2 months
Large Munsterlander	F	10 years 7 months
Lhasa Apso	FN	11 years 7 months
Lhasa Apso	FN	11 years 7 months
Miniature Schnauzer	FN	10 years 1 months
Sharpei	F	6 years 9 months

### Sequencing

Mononuclear cells were isolated from the peripheral blood using Ficoll-Paque (GE Healthcare) following the manufacturer’s instructions. The cells were processed into mRNA using polyA-pulldown, sheared, and sequenced on a HiSeq 2500 machine (Illumina) using 250 bp paired-end reads by the core sequencing team at the Wellcome Trust Sanger Institute. Illumina HiSeq paired-end sequencing reads were aligned to the *C. lupus familiaris* reference genome CanFam3.1 using GSNAP version 2015-11-20 and alignments viewed and annotated with Otterlace (Searle 2004; Wu and Nacu 2010).

### Gene naming

AR V genes were divided into subgroups based on IMGT criteria and assigned as functional, pseudogenes, or ORF using the same criteria as Bao et al. (2010). Briefly, a V gene was classed as functional if it had no frameshifts or in-frame stop codons and included a conserved tryptophan and two conserved cysteines. An otherwise functional V gene that lacked any of the three conserved amino acids was classed as an ORF, and all other genes were deemed pseudogenes. Subgroup numbers were assigned based on homology to human subgroups, and given a new number in the cases where no obvious match could be found. All gene names were assigned in keeping with the nomenclature system of IMGT, and all annotations will be available from the IMGT databases and tools (Lefranc et al. 2009).

### V gene comparisons

Functional V genes from the human, dog, and mouse were identified and grouped into IG or TR. Within these two groups, all the germline nucleotide sequences were aligned using Clustal Omega (McWilliam et al. 2013). The maximum identity score from one species to each of the two others was plotted. The maximum score was chosen as the different gene subgroups have grown and changed at different rates across the species and so one to one comparison is not numerically possible across the genes. The identity scores were compared and the confidence of the differences was assessed by the Student’s *t* test.

### Non-reference alleles

Variant call files mapping to the AR loci from 107 canine whole genome sequences (Table 2) were kindly provided by Steven Friedenberg from the University of Minnesota. Alleles for annotated immunoglobulin and T cell receptor genes were extracted from the VCF (Variant Call Format, http://www.htslib.org/doc/vcf.html) files using bcftools version 1.2 of the samtools suite of programs (http://www.htslib.org) run with the option ‘consensus -H’ (Li et al. 2009). For clarity, the sequence found in the CanFam3.1 genome build is referred to throughout this paper as the reference allele, and all the new references identified in this variant call file dataset are referred to as novel or non-reference alleles.

**Table 2:** Number and breeds of the dogs within the 107 whole genome sequences. Both chromosomes were represented for each dog

Breed	Number
American Staffordshire	1
Boxer	22
Cavalier King Charles Spaniel	10
Collie (Smooth)	4
Dachshund (Smooth)	3
Doberman Pinscher	4
German Shepherd	1
Golden Retriever	2
Great Dane	6
Irish Setter	3
Miniature Poodle	1
Rhodesian Ridgeback	3
Scottish Deerhound	5
Scottish Terrier	6
Shetland Sheepdog	1
Standard Poodle	20
Toy Poodle	3
West Highland White Terrier	4
Yorkshire Terrier	8
Sum	107

For the incidence of unique alleles, the two-sample Kolmogorov-Smirnov test was used to determine whether any breed had a significantly different number of unique alleles from the others. The Bonferroni method was applied as a multiple comparisons correction.

### Inter- and intra-species loci alignments

Sequences were masked using RepeatMasker and alignment plots generated using PipMaker (Smit et al.; Schwartz 2000).

### Phylogenetic analysis

Nucleotide sequences were aligned using Clustal Omega and the output tree was visualised using the Interactive Tree of Life (Letunic and Bork 2007; McWilliam et al. 2013).

## Results

### Gene numbers

**Fig. 1:**
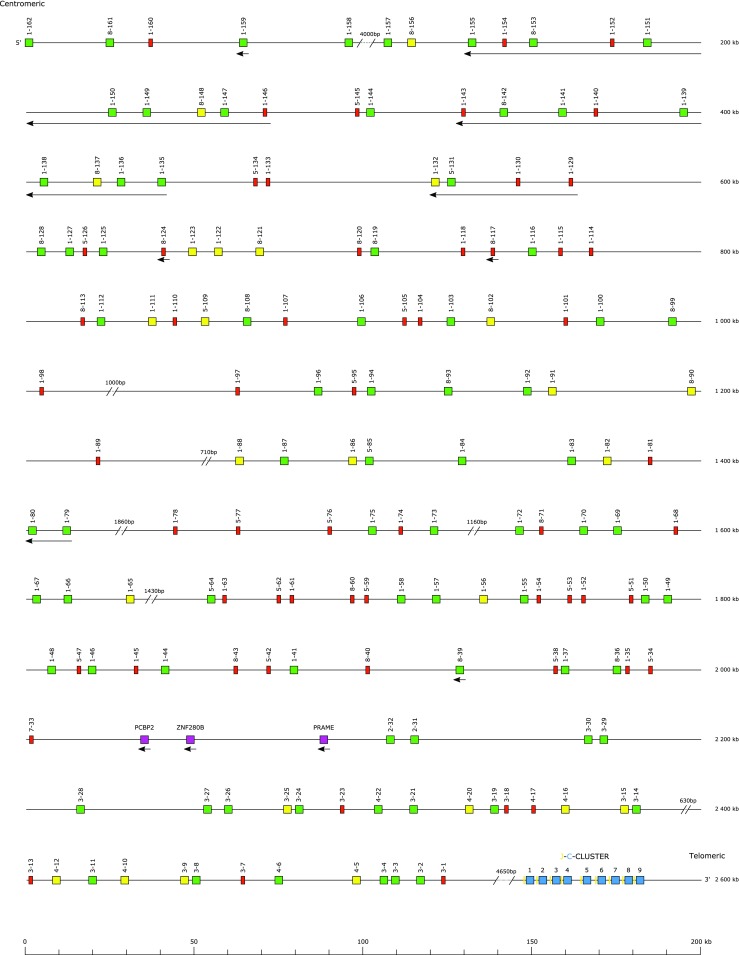
The canine immunoglobulin lambda locus

**Fig. 2:**
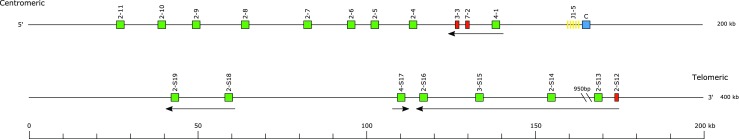
The canine immunoglobulin kappa locus

The largest number of new annotations was in the immunoglobulin light chain loci (Table 3). A total of 162 IGLV genes across seven gene subgroups were identified, of which IGLV1 was the largest with 86 members (Fig. 1). In keeping with other IGL loci, the J and C genes were found as pairs, nine in total. Nineteen IGKV genes were identified, of which 14 were from the IGKV2 subgroup, along with 5 IGKJ and 1 IGKC gene (Fig. 2).

**Table 3:** Count of genes, by gene type and locus, for each of the canine loci. NB—the TRD locus is located within the TRA locus and as such expressed TRD chains may include a TRAV

		Locus							
		IGH	IGK	IGL	TRA	TRB	TRD	TRG	Sum
Gene type	V	83	19	162	39	38	5	16	362
	D	6				1	2		9
	J	6	5	9	59	6	3	16	104
	C	22	1	9	4	4	5	27	50
	Sum	117	25	180	102	49	15	59	547

**Fig. 3:**
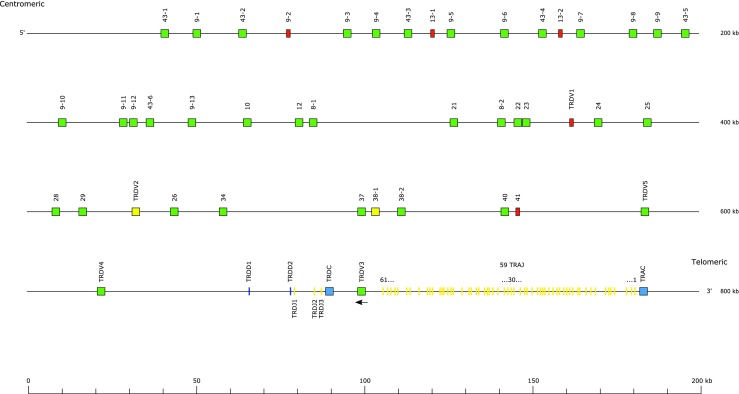
The canine T cell receptor alpha/delta locus

A total of 44 V genes were assigned to the TRA/TRD locus, with 5 being definitely marked as TRDV (Fig. 3). There were two TRDD, three TRDJ, one TRDC, and one TRAC gene identified. A total of 59 TRAJ genes were annotated, bearing the same level of homology to, and therefore being numbered in the same way as, the 60 mouse TRAJ genes compared to the 61 human TRAJ genes.

**Fig. 4:**
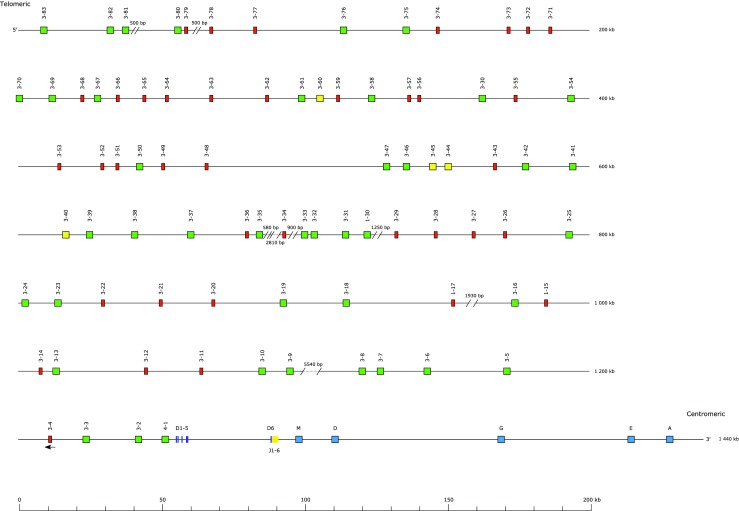
The canine immunoglobulin heavy chain locus

Within the previously annotated IGH, TRB, and TRG loci, four new TRBV genes (TRBV4-4, and TRBV31 to TRBV33) and three new IGHJ genes (IGHJ1, IGHJ2, and IGHJ5) were identified (Fig. 4).

### Loci structures

The canine IGH locus is located just sub-telomeric of chromosome 8, on the antisense strand. This telomeric location and orientation is observed in all mammals with the exception of monotremes and marsupials (Das et al. 2008). The light chain and TRA/TRD loci, however, do not show strong conservation of chromosomal location between human, mouse, and dog.

The canine IGH locus has all functional genes transcribed in the same sense as the constant regions, with one pseudogene (IGHV3-4) in the reverse transcriptional orientation (Fig. 4). The human and mouse IGH loci are similar in this respect. The overall structure of the IGH locus is broadly similar to that described by Bao et al. (2010); however, three new IGHJ genes have been identified. There are small differences in the position and expected functionality of the V genes, although absolute ratios and numbers match. The discrepancy may be due to the use of different builds of the reference genome, with this annotation using the most up to date publically available build (CanFam3.1).

The canine IGK locus is both small (400 kbp) and has an unusual structure (Fig. 2). It has 11 V genes, 9 of which are functional, upstream of the J and C genes, as well as 9 genes downstream, of which 8 are functional, and inverted with respect to the J and C genes. Thirteen of the 16 functional genes are members of the IGKV2 subgroup, 2 are members of the IGKV4 subgroup (1 in the upstream cluster and 1 in the downstream cluster) and just 1 gene is a member of the IGKV3 subgroup in the downstream cluster. Curiously, both IGKV4 genes are in an inverted orientation with respect to the rest of the genes in the upstream and downstream clusters. The inverted structures are reminiscent of the equine IGL locus, where it was found that orientation had no impact on V gene usage (Hara et al. 2012).

Inversions and block duplications appear to be a feature of light chain loci, in particular IGK loci. Not only do the IGK loci of humans, pigs, mice, horses, and dogs all contain V genes with the opposite transcriptional orientation to the C gene, but the dog, human, and pig loci have undergone inversional duplication of entire blocks (Schwartz et al. 2012a; Walther et al. 2015). In the pig and the human loci, the genes in the two blocks have diverged sufficiently little that some or all are known by the same gene ID as their pair in the other block (Kawasaki et al. 2001; Schwartz et al. 2012a).

The canine IGL locus is large (2.6 Mbp) with 162 V genes, of which 78 are functional. Many V genes are inverted with respect to the J-C cluster (Fig. 1), a feature not seen in humans, pigs, or mice (Schwartz et al. 2012b). These inversions appear to be or have been under a degree of location-specific selection pressure, with only 3 of the 116 most C-proximal IGLV genes in the opposite transcriptional orientation to the J-C genes, whilst 26 of the final 46 V genes are inverted. This partly reflects block duplications of clusters of inverted IGLV1 and IGLV8 genes whose members show high levels of sequence identity in a pattern that is positionally conserved within repeating blocks.

The canine IGH locus has a gene to pseudogene ratio of 1:1, which is consistent with other known IGH loci, and equates to proportionately more pseudogenes than the ~ 4:1 ratios typically found in light chains (Das et al. 2008). In most species studied to date, the ratio of functional genes between the IGK and IGL loci correlates with the relative usage of each chain (Arun et al. 1996; Sun et al. 2013). For example, the mouse is an IGK-biased species. Its IGL locus only contains nine functional genes, which mirrors its use in only 5% of expressed antibodies (Sun et al. 2013). In contrast, the dog is an IGL-biased species, with 91% reported usage of the IGL chain, which reflects the relative sizes of its light chain functional repertoires (Arun et al. 1996).

The TRA/TRD locus has a similar structure to those of the human and mouse (Fig. 3). The TRDD, TRDJ, and TRDC genes are flanked by TRDV genes, with the downstream TRDV inverted with respect to the rest. This block lies upstream of a large cluster of TRAJ genes and the TRAC gene. Phylogenetic analyses show the high degree of homology between the dog, mouse, and human TRAJ genes (data not shown) in a way that mirrors previous findings (Koop and Hood 1994). In a manner reminiscent of the dog IGL locus, the C-distal end of the locus appears to have undergone block duplications, with repeating units of TRAV9, TRAV43, and TRAV13 genes found there and nowhere else in the locus.

### Inter- and intra-species loci alignments

**Fig. 5:**
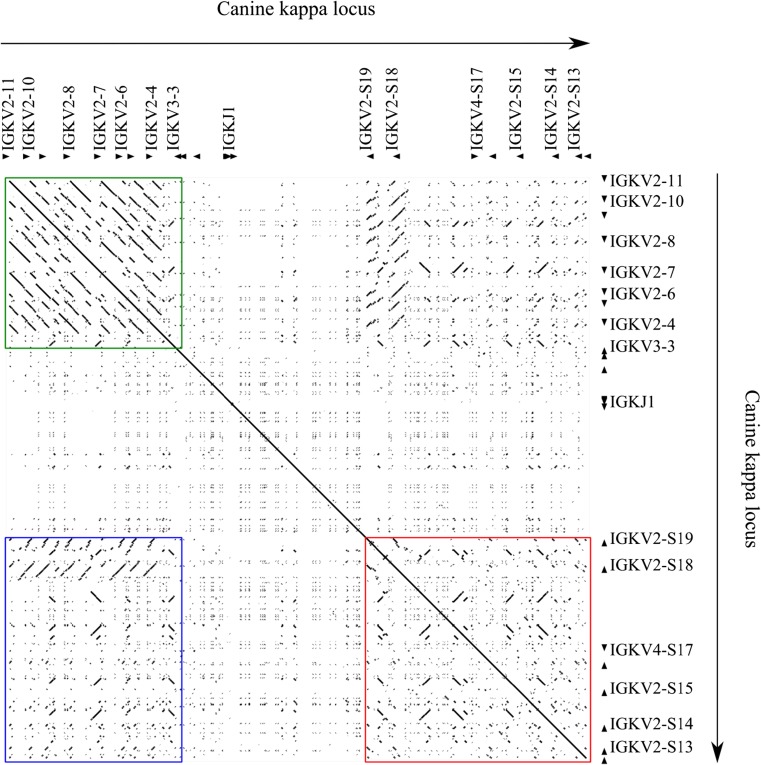
Self-alignment of the canine immunoglobulin kappa locus. The green box represents the upstream V genes aligned to themselves. The red box represents the downstream V genes aligned to themselves. The blue box represents the alignment of the upstream V genes against the downstream ones

Alignments were carried out with PipMaker using the canine IGK, IGL, and TRA/TRD loci against themselves and against their respective mouse and human loci (Schwartz 2000). In the IGK self-alignment, three comparisons are of note: the upstream and downstream blocks’ self-alignment (Fig. 5 green and red boxes, respectively), as well as their alignment with each other (Fig. 5 blue box). The solid lines off the main diagonal indicate likely duplication events, with the gaps representing mutations accumulated since the duplication.

The multiple broken diagonals in the upstream self-alignment are characteristic of a block, in this case a single V and its flanking sequence, being locally copied multiple times, similar to the block duplication of a three-gene cassette in the canine TRB locus (Mineccia et al. 2012). In the self-alignment of the downstream block, the lines are shorter and at times perpendicular, implying local inverted homology. Finally, the homology comparison of the upstream to the downstream block reveals a good degree of homology, particularly IGKV2S18 and IGKV2S19 to the IGKV2 genes upstream of the C gene. This pattern would be seen where a single gene replicated upstream of the C gene multiple times, then a block inversional duplication occurred in a manner reminiscent of human IGK and equine IGL. This downstream block was then likely under reduced selection pressure and has accumulated mutations and local inversions at a greater rate, with the exception of the IGKV2S18 and IGKV2S19 genes, which have diverged less. Whilst this is not the only possible explanation, it is consistent with proposed explanations for similar features in AR loci of other species.

**Fig. 6:**
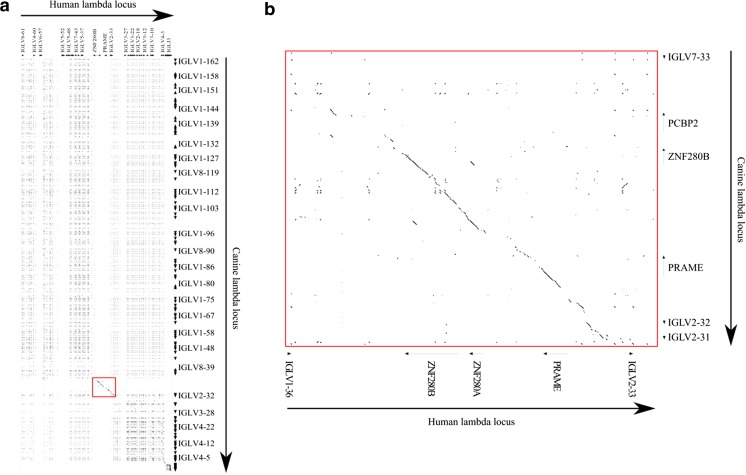
The alignment of the canine and human immunoglobulin lambda loci. **a** The complete loci. **b** An amplified section of the alignment, corresponding to the red box in **a**

In the comparison of the dog and human IGL loci, a high degree of homology is apparent near the midpoint of the human locus (Fig. 6a). This region spans the region of the human locus that includes the non-AR genes ZNF280A, ZNF280B, and PRAME (Fig. 6b). The sequences for ZNF280B and PRAME and most of the surrounding region are highly conserved between dog and human, implying that they are likely to be of similar functional importance. ZNF280A can neither be reliably identified at this locus nor elsewhere in the canine genome (data not shown); however, another non-IGL canine gene (PCBP2) is present near ZNF280B.

Non-AR genes interspersed among the AR loci is a common feature across species, although the locations and genes in question are not always conserved. For example, ADAM6 genes are found in the IGH loci, in between the IGHV and IGHD genes of human and mouse, although the human orthologue is non-functional. Whilst no orthologue has been characterised in dog, there are two candidates: one of which is found between IGHV3-4 and IGHV3-5, and the other is upstream of the IGH locus entirely. Given the limited knowledge of the ADAM gene family in dogs, this potential orthologue has not been added to the IGH annotation. No other non-AR genes have been identified in the canine IGK, IGL, or TRA/TRD loci.

**Fig. 7:**
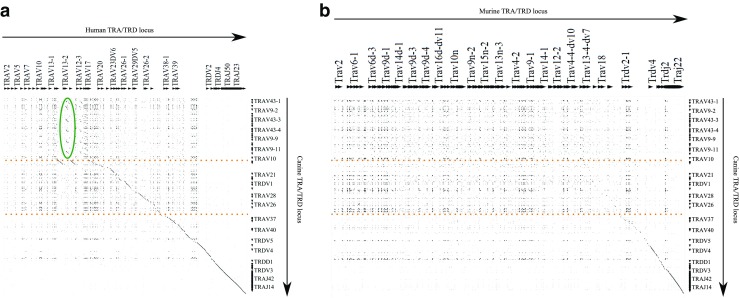
Alignment of the canine TRA/D locus to **a** the human locus and **b** the mouse locus. Orange dashed lines represent boundaries between homology regions. The green oval highlights evidence for a block duplication in the canine locus

When considering the TRA/TRD D percentage identity plots, the similarity across dog comparisons to human and to mouse is of note (Fig. 7a, b). Comparisons between the human and mouse TRA/TRD loci have been described, and the ‘striking sequence similarity’ at the J and C end of the locus was noted (Koop and Hood 1994; Glusman et al. 2001). With the canine comparisons, there is equivalent homology, running from the 3′ end of the locus to near TRAV37 for the mouse (Fig. 7b lower orange dashed line), and TRAV10 for the human (Fig. 7a upper orange dashed line). This extended homology is reminiscent of the homology between canine TRG and its human counterpart (Massari et al. 2009). There are also the characteristic regions of homology between each V gene across the species, seen most readily above the lower orange dashed line (Fig. 7a, b). The comparison to human also lends weight to the proposed block duplications at the C-distal end of the locus as there are repeated long blocks of homology that span multiple genes (Fig. 7a green oval).

### V gene comparisons

**Fig. 8:**
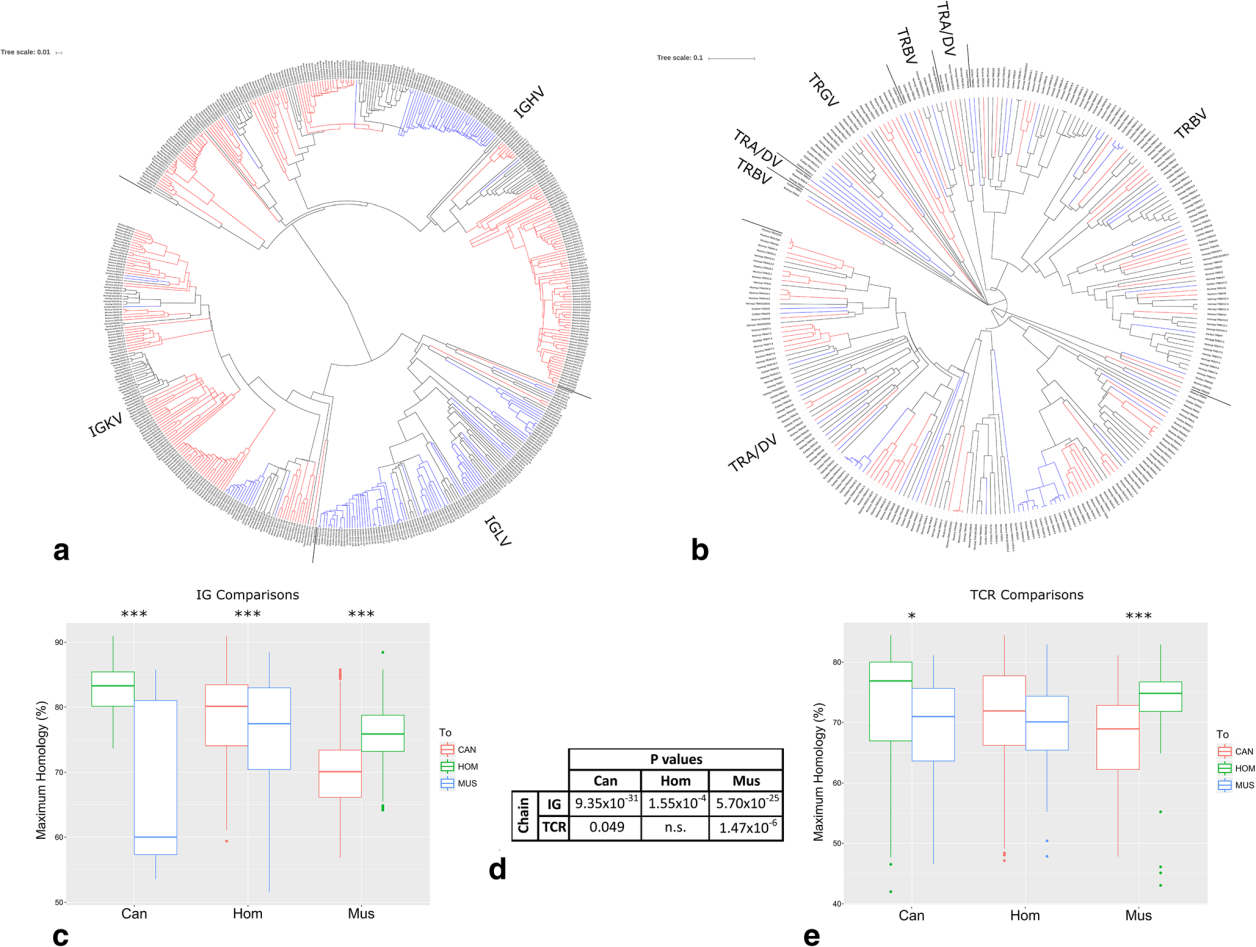
AR V gene comparisons between dog, human, and mouse. Phylogenetic tree of all of the functional **a** IG V and **b** TR V genes in human (black), dog (blue), and mouse (red). Plot of the maximum individual homology of each **c** IG V and **e** TR V gene from dog (red), human (green), and mouse (blue) to the nearest gene from each of the other two species. Student’s *t* test was carried out within each comparison, and the *p* values are shown (**d**)

Whilst the full loci alignments do lend insight into the evolutionary background of these genes, direct comparisons allow for quantitative assessment of these relationships. Phylogenetic trees were constructed of the functional IG (Fig. 8a) and TR (Fig. 8b) V genes from human, mouse, and dog. The IG V genes segregated by chain, but there was a degree of interspersion among the TR V genes, particularly some of the TRA/DV genes. With the IG V genes, the dog and human genes appear to be the most closely related, but the relationship is less clear in the TR genes.

To quantify this relationship, each V gene within the IG (Fig. 8c) and TR (Fig. 8e) groups was aligned and compared to every V gene from the other two species. These comparisons highlight the greater divergence seen in the TR loci compared to the IG loci, as shown by the maximum homology scores being generally lower, a result consistent with previous work (Stewart et al. 1997). Within the IG loci, the dog and human are most similar to each other, regardless of the direction of comparison, and the mouse is more similar to human than it is to dog. The TR loci, however, show a less defined pattern. The dog and the mouse TR V genes are both more similar to human than either species is to each other, whilst the human TR V genes are not significantly more similar to either (Fig. 8d). This may reflect the constraints imposed by co-evolution with gene families with which the TRs interact, such as the MHC locus (Olivieri et al. 2013).

### Non-reference alleles

Whole genome sequences from 107 dogs from 19 breeds, all aligned to the current reference build, were interrogated for novel AR alleles based on the nucleotide sequence, and 5516 were identified across the six loci (Table 4). As can be expected, there were many more new V gene alleles found than new alleles for any other D or J gene, and in a manner that broadly reflects the size of each locus. In terms of allele distribution across all genes, the reference allele was found 89,355 times out of the 117,058 alleles called (76%). No significant breed specificity in allele distribution was identified.

**Table 4:** Count of novel non-reference alleles identified from 107 whole genome sequences

		Locus							
		IGH	IGK	IGL	TRA	TRB	TRD	TRG	Sum
Gene type	V	1723	107	2347	776	132	11	75	5171
	D	7							7
	J	12		1	9	1		7	30
	C	180	1	68	4	3	13	39	128
	Sum	1922	108	2416	789	136	24	121	5516

In the same manner as the reference allele, non-reference alleles were denoted as functional, pseudogene, or ORF, in accordance with the IMGT standard (Lefranc et al. 2009). Given that the mutation(s) that distinguish the new allele from the reference could introduce a frameshift, or otherwise alter functionality, the relative functionality of the new allele was noted. The majority (72.8%) of the new alleles have the same functionality as the reference allele (Table 5).

**Table 5:** Functionality comparison of non-reference V alleles to their reference allele. Novel V alleles were defined as F, ORF, or P and this was compared to the original allele. The type of change is represented in the Change column, and the relative proportions of each are in the Fraction column

Class	Change	Fraction	Sum
Loss	F->ORF	4.61%	23.78%
	F->P	14.29%	
	ORF->P	4.89%	
Gain	ORF->F	0.70%	3.37%
	P->F	1.20%	
	P->ORF	1.48%	
None	PP	13.69%	72.85%
	FF	50.50%	
	OO	8.66%	

As the reference allele is found so frequently across the different genes of each locus, and this is seen in all of the dogs in the sample, this strongly indicates that it is the ancestral allele and predates breed divisions. The implication of this is that in the rare instances where a novel allele is found, it represents the novel mutant, and therefore a change that has occurred under selection. Considering the nine permutations of relative functionality between novel and reference, it is possible to cluster them based on the direction of that change. Novel alleles that were less functional than the reference are grouped as ‘Loss’ and occurred 23.8% of the time, whilst those that were more functional make up the remaining 3.4% of allele changes.

**Fig. 9:**
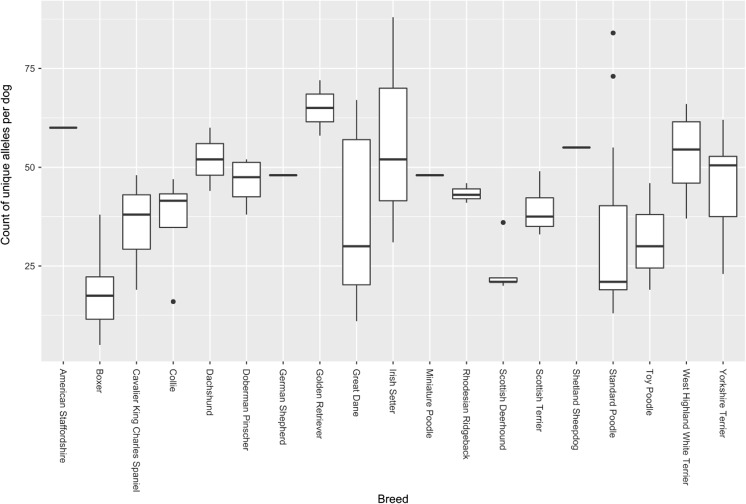
Incidence of unique alleles. The number of unique alleles per dog is plotted per breed, where a unique allele is a non-reference allele found only once in a single dog within the dataset

Non-reference V alleles were called 21,704 times across all samples and loci. Interestingly, IGKV2S13, TRBV2-1, and eight IGLV genes were only found as non-reference alleles, including in the boxer samples, noteworthy as the reference genome is from a boxer. Considering the other boxer samples in the dataset, the average number of unique V alleles per dog was 18, with a range of 5–38 (Fig. 9). Pairwise comparisons of each breed to all others were carried out and only the boxer was found to have a significantly different distribution of unique allele counts relative to the rest of the dataset (*p* < 0.000001). Given that the denomination of non-reference is relative to a boxer genome, it is not surprising that the only instance of a breed being compared to itself is different from all other breeds. In other words, the result confirms that the breeds are reasonably clustered within themselves, and that overall there is not a large variation between them in terms of unique alleles. The ten unique alleles within the reference, therefore, likely represent non-ancestral alleles within the dog that was used to build the reference genome, rather than being representative of the species as a whole. As such they were not included in the subsequent analysis, although the findings remained the same when they were included (data not shown).

Considering the number of times a non-reference allele was called within the dataset, working from the assumption that they represent changes from the ancestral gene, inferences can be drawn about selection pressures. If a gain or loss of function of a given gene had no impact on fitness, then the 21,704 times a novel V was found should reflect the distribution of the non-reference alleles themselves. For example, 3.4% of the 5171 novel alleles are ‘Gain’, so one would expect Gain alleles to be found 738 times in the dataset as a whole.

**Fig. 10:**
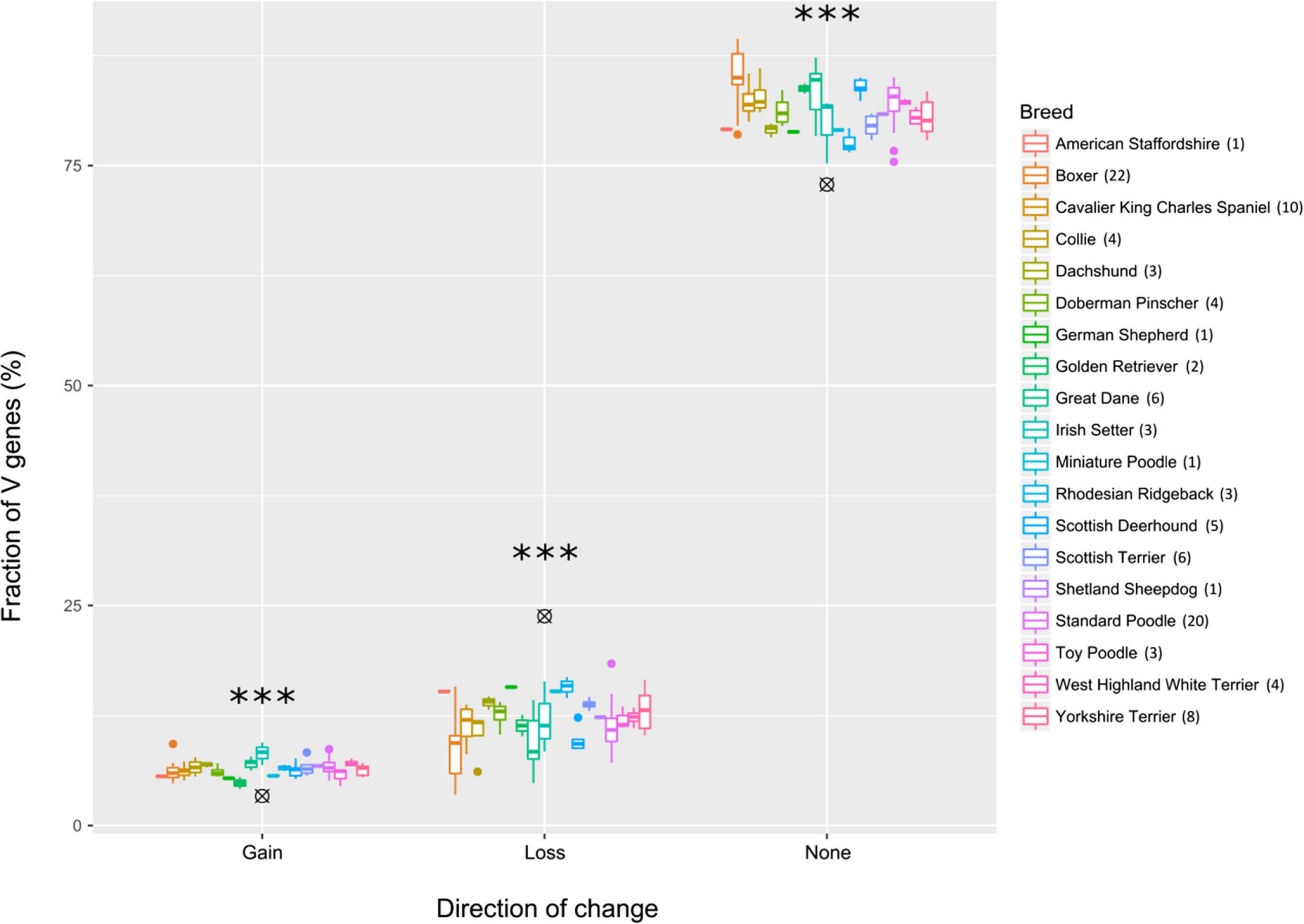
Allele changes. The proportion of non-reference V gene alleles is plotted per breed based on three classes. Alleles that were functional where the reference allele was not, as well as those that were ORFs where the reference was a pseudogene, are grouped as ‘Gain’. Alleles that were pseudogenes where the reference allele was not, as well as those that were functional and became ORFs, are grouped as ‘Loss’. Alleles with no functionality difference are classed as ‘None’. Black crosshairs represent the expected values for each change type and the number of samples per breed is in parentheses after the breed type

However, Loss changes are found less often than expected, and no change and Gain are found more often than expected (Fig. 10). Furthermore, the variances were very low across the breeds, lending further weight to the selection pressures on the AR loci having limited breed-dependence. A *z* test was carried out and the differences between the population and expected means were highly significant, ranging from *p* = 2.34 × 10^−25^ down to *p* < 1 × 10^−250^.

## Discussion

### Evolutionary insights

Dogs exhibit high levels of inter-breed heterogeneity and intra-breed homogeneity. It has been estimated that breed formation has accounted for a 35% loss in nucleotide diversity (Gray et al. 2009). Recent work identified 22 blocks of homozygosity longer than one megabase in certain breeds, which the authors attributed to breeder-imposed selection pressure (Vaysse et al. 2011). This contrasts with the diversity seen in humans where the separation between even the least related populations only explains 5–10% of their variation, whereas more than one quarter of the genomic variation in dogs is attributable to breed, not individual, variation (Parker 2012).

Given the level of breed specificity of canine genomes, including a high level of specificity in MHC alleles, it is perhaps surprising that the non-reference AR alleles did not exhibit strong breed-specific haplotypes. However, this should be considered in context. Domestication of dogs from wolves occurred around 15,000 years ago, but most breeds have only been formed in the last few hundred years from very few individuals per breed, and typically with only minimal genetic input from outside the breed thereafter (Vaysse et al. 2011). The MHC loci contain far fewer genes than the AR regions, but there are more alleles at each locus, so that the effects of bottlenecking at breed foundation are much more apparent in the MHC.

Whilst the concerted efforts of breeders have managed to yield a great deal of diversity in comparatively few generations around loci at the focus of selective sweeps, there is no a priori reason for great breed divergence at loci distant from those selected for, either intentionally or passively. A thorough comparison to wolves or wild dogs would be required, but if it is indeed the case that the reference is representative of the AR genes of the species and its ancestral genome then it is because these are loci as yet unaffected by breed formation. This is unlikely to be a unique feature of AR loci, and may have clinical implications, namely verifying whether therapeutic targets do or do not vary to an extent that may impact efficacy.

Moving beyond the differences that breeds have with each other, the phylogenetic comparisons of canine, murine, and human AR genes indicate a closer genetic relationship between humans and dogs than mice with either. Whilst this does appear to go against the established phylogeny of laurasiatheria versus the euarchontoglires, there have been phylogenomic studies that have called for a revision of this relationship (Cannarozzi et al. 2007; Elhaik and Graur 2014). It is also not the first time that human proteins were found to have closer homologues in dogs than mice as a number of key cancer-related genes were found to have greater sequence identity with dog than with mouse (Cekanova and Rathore 2014). Whilst this work does not seek to make definitive claims as to overall phylogeny, it does add to a body of research making a case for the relative benefits of dogs over mice as a model organism, in this case in immunology (Cekanova and Rathore 2014; Anderson and Modiano 2015; Hayward et al. 2016).

### Allelic distributions

The two best represented breeds in this data set were the boxer and standard poodle (22 and 20 dogs, respectively). A small number of non-reference alleles were found relatively frequently in both breeds, whilst rare alleles were typically only found on a single chromosome of one dog from one breed. Less well-represented breeds followed similar distributions, and given the number of singularly represented alleles, it seems that non-reference alleles are typically heterozygous with the reference one.

It is possible that larger breed-specific cohorts may reveal trends not apparent in this dataset, but we conclude that the selective pressures that have shaped the AR gene loci are breed-agnostic. There are a limited number of examples where less common alleles are frequently found in specific breeds, which likely reflect breed-specific population bottlenecks or alleles that have evolved since breed foundation. For example, IGLC1*01 (the reference allele) is found on 19 of the 44 boxer chromosomes sequenced, and 1 of the 6 toy poodle chromosomes, but is not found in any other breeds. One hundred ninety-four of the 214 called alleles across all breeds are represented by the other allele identified, IGLC1*02. But given that all of the breeds in this sample, with the previously-discussed exception of the boxer, show distributions of unique alleles that are not significantly different from those of all others, it is likely that this is representative of novel alleles of all incidences.

The distributions of these non-reference alleles are all broadly as would be expected. A large and diverse V gene repertoire generally offers the organism a fitness advantage; hence, there is a bias towards gain and away from loss of function. However, not all V genes will confer an advantage as some could be auto-reactive or offer no improvement on existing alleles. This is one of the likely drivers behind no change occurring more frequently than expected as beneficial genes are not lost, and deleterious ones are not activated.

That gain of function is more common than expected lends weight to theories put forward regarding the high pseudogene load of the AR loci. Pseudogene loads are generally high in AR loci compared to other gene families, particularly in the dog, and are often expressed (Kawasaki et al. 2001; Das et al. 2008; Hara et al. 2012; Mineccia et al. 2012). They can gain functionality in recombination, for example where a stop codon is lost due to SHM or recombination itself, and act as a substrate for gene conversion (Sun et al. 2012). Assuming that the reference allele is the original, which is in line with their near ubiquity, the non-reference alleles that are more functional than their parent allele are examples of the ORF and pseudogenes being a mutable starting pool for new beneficial alleles and are under a selection pressure as such.

## Conclusions

With this work, the dog becomes only the third species, after man and mouse, to have all of its AR loci sequenced. This, combined with the large numbers of alleles identified, provides an excellent resource for use in canine immunology as well as immunogenetics more broadly. Furthermore, their close homology with humans reinforces the dog as an excellent immune model.

Overall this work forms the basis for functional analysis of the AR loci, both in the healthy dog with comparison to the human system, and in disease states in order to better understand and treat canine pathologies.
